# State Estimation for Quadruped Robots on Non-Stationary Terrain via Invariant Extended Kalman Filter and Disturbance Observer

**DOI:** 10.3390/s24227290

**Published:** 2024-11-14

**Authors:** Mingfei Wan, Daoguang Liu, Jun Wu, Li Li, Zhangjun Peng, Zhigui Liu

**Affiliations:** 1 College of Information Engineering, Southwest University of Science and Technology, Mianyang 621010, China; liudaoguang@mails.swust.edu.cn (D.L.); pzj1@swust.edu.cn (Z.P.); 2 Mianyang Zhongke Huinong Digital Intelligence Technology Co., Ltd., Mianyang 621010, China; wujun@swust.edu.cn; 3 Sichuan Engineering Technology Research Center of Industrial Self-Supporting and Artificial Intelligence, Mianyang 621010, China; ll@swust.edu.cn; 4 School of Computer Science and Technology, Southwest University of Science and Technology, Mianyang 621010, China; 5 School of Life Science and Engineering, Southwest University of Science and Technology, Mianyang 621010, China

**Keywords:** quadruped robots, invariant extended Kalman filter, state estimates, non-stationary terrain

## Abstract

Quadruped robots possess significant mobility in complex and uneven terrains due to their outstanding stability and flexibility, making them highly suitable in rescue missions, environmental monitoring, and smart agriculture. With the increasing use of quadruped robots in more demanding scenarios, ensuring accurate and stable state estimation in complex environments has become particularly important. Existing state estimation algorithms relying on multi-sensor fusion, such as those using IMU, LiDAR, and visual data, often face challenges on non-stationary terrains due to issues like foot-end slippage or unstable contact, leading to significant state drift. To tackle this problem, this paper introduces a state estimation algorithm that integrates an invariant extended Kalman filter (InEKF) with a disturbance observer, aiming to estimate the motion state of quadruped robots on non-stationary terrains. Firstly, foot-end slippage is modeled as a deviation in body velocity and explicitly included in the state equations, allowing for a more precise representation of how slippage affects the state. Secondly, the state update process integrates both foot-end velocity and position observations to improve the overall accuracy and comprehensiveness of the estimation. Lastly, a foot-end contact probability model, coupled with an adaptive covariance adjustment strategy, is employed to dynamically modulate the influence of the observations. These enhancements significantly improve the filter’s robustness and the accuracy of state estimation in non-stationary terrain scenarios. Experiments conducted with the Jueying Mini quadruped robot on various non-stationary terrains show that the enhanced InEKF method offers notable advantages over traditional filters in compensating for foot-end slippage and adapting to different terrains.

## 1. Introduction

Quadruped robots, which draw inspiration from four-legged animals, are advanced robotic systems. In contrast to other mobile robot types, quadruped robots possess outstanding stability and flexibility. They are able to traverse a wide variety of complex and uneven terrains, including sand, grass, gravel roads, and even slippery surfaces. These capabilities endow quadruped robots with great potential for applications in rescue missions [1], environmental monitoring [2], and smart agriculture [3]. As they are increasingly used in challenging scenarios such as mountainous areas, forests, and post-disaster rubble environments, precise state estimation becomes crucial for their stability and reliability.

Multi-sensor fusion-based state estimation algorithms are commonly used in quadruped robots. These algorithms incorporate external sensors like LiDAR, cameras, and GPS, along with proprioceptive sensors such as IMUs and joint encoders [4]. Although external sensors supply abundant environmental information, they do have limitations. For instance, cameras exhibit poor performance in low-light or low-texture environments, the accuracy of LiDAR deteriorates under smoky or dusty conditions, and GPS signals can be disrupted in indoor or urban canyon settings [5]. In contrast, proprioceptive sensors, which rely on joint kinematics and IMU data, are not affected by such environmental factors and provide higher measurement frequencies. This enables the fast and precise control of quadruped robots. Consequently, it is of vital importance to explore state estimation methods based on proprioceptive sensors like IMUs and joint encoders. IMUs provide high-frequency acceleration and angular velocity data, while joint encoders offer precise measurements of joint angles and speeds. The combination of these sensors allows for accurate motion state estimation in complex environments, especially when external sensors malfunction or are limited. This approach augments the autonomy, robustness, and reliability of quadruped robots in various scenarios.

State estimation methods for legged robots date back to 2005 when Lin et al. [6] developed an approach based on leg kinematic information for a hexapod robot. This method required the robot to maintain a tripod gait with three legs always in contact with a flat terrain. In 2006, Lin et al. [7] further proposed fusing leg odometry with IMU data to improve state estimation during tripod gait running. Since then, state estimation for legged robots has garnered significant attention. Bloesch et al. [8] introduced a quadruped robot state estimation method using an extended Kalman filter (EKF), incorporating leg contact points into the filter’s state variables and using forward kinematics to estimate leg contact positions and body pose. Camurri et al. [9] developed a legged odometry method without contact sensors, using ground reaction forces to determine the reliability of leg contact and weigh each leg’s contribution to body velocity. However, due to inconsistencies between EKF’s positive feedback and observation information, the filter may diverge. To address this, Hartley et al. [10] proposed a contact-aided invariant extended Kalman filter (InEKF) based on invariant observer theory, showing better performance compared to the traditional quaternion-based EKF.

The aforementioned methods assume stable, slip-free foot-end contact between legged robots and the ground. However, on slippery or soft terrains such as grass, mud, or sand, foot-end slippage occurs, rendering stable contact a challenge. This leads to the introduction of non-Gaussian errors that accumulate over time and cause unbounded drift. To tackle this issue, Ting et al. [11] regarded slippage in foot-end observations as an outlier and used a weighted least squares method to mitigate its impact on the EKF. Similarly, Bloesch et al. [12] replaced the EKF with an unscented Kalman filter (UKF) and applied an outlier rejection method to manage occasional slippage. Jenelten et al. [13] developed a probabilistic slip detector using a Hidden Markov Model, enabling robots to walk on slippery surfaces through impedance control and friction adjustment. However, this approach does not completely resolve pose estimation drift in pose estimation. To reduce drift caused by slippage, some methods fuse external sensor observations. Wisth et al. [14] proposed a state estimation method based on factor graph optimization that tightly integrates visual features, inertial data, and kinematic constraints. This method was further extended to incorporate LiDAR observations and an online-estimated linear velocity deviation term to minimize drift in legged odometry [4]. Kim et al. [15] introduced a state estimator for quadruped robots based on a pre-integrated foot velocity factor, which does not rely on precise contact detection or fixed foot positions, showing strong performance on uneven and slippery terrains. However, factor graph-based methods use measurements along the entire trajectory for smoothing, which limits their update rates for real-time control. To address this limitation, Teng et al. [16] integrated camera observations into the invariant extended Kalman filter (InEKF) to enhance state estimation on slippery terrains. Fink et al. [17] combined a Global Exponential Stability (GES) nonlinear attitude observer with legged odometry, ensuring consistently bounded speed estimation and reducing drift in unobservable position estimates.

Recently, advancements in computational hardware (e.g., GPUs) have enabled the training and deployment of complex deep learning models, encouraging researchers to develop learning-based slip detection and state estimation methods. Rotella et al. [18] proposed a method using fuzzy clustering to learn contact probabilities for humanoid robots. When integrated into a basic state estimation framework, this method can significantly reduce estimation errors. Buchanan et al. [19] developed a deep neural network to learn motion patterns from IMU data. When combined with traditional legged odometry, it substantially reduces drift in proprioceptive state estimation. Yang et al. [20] applied neural networks to train weight functions for foot-end forces and legged odometry states, enhancing observation accuracy. However, these methods also face their own technical challenges. Supervised models require large amounts of labeled data, which is extremely difficult to obtain in practice. Additionally, unsupervised methods, which treat slip detection as a classification task, limit the ability to observe slip velocity. This severely affects the performance and generalization ability of the models and is a technical bottleneck that needs to be overcome when applying such methods to quadruped robot state estimation.

Actually, unstable foot-end contact can be regarded as a form of deviation within the filter framework, as it introduces non-Gaussian errors that accumulate over time and lead to unbounded drift in state estimation. In state estimation systems where biases affect the measurements or system dynamics, researchers have developed several advanced Kalman filter-based approaches to address these issues. Zhong et al. [21] demonstrated the effectiveness of a UKF in vehicle state estimation by handling environmental biases, such as those caused by slope and wind conditions. Bellés et al. [22] developed robust error-state Kalman filters that incorporate error states, significantly improving resilience against non-Gaussian disturbances. In another approach, Huang et al. [23] proposed a robust Kalman filter that utilizes an adaptive estimation of time-varying measurement bias specifically designed to handle unknown, non-Gaussian, heavy-tailed noise through a Student’s t–inverse-Wishart distribution. This method provides an important reference for addressing complex noise conditions in state estimation. Additionally, Zhang et al. [24] proposed a distributed bias-correcting estimator, which independently adjusts each sensor’s bias, providing scalability and robustness in complex environments with persistent biases. Inspired by those studies, we consider modeling slip velocity as a deviation term of speed and explicitly incorporate it into the state equations to reduce the pose drift caused by slip through slip velocity estimation. To meet real-time requirements, we opted for a filtering-based method. The invariant extended Kalman filter (InEKF), developed based on invariant observer theory, has been successfully applied in simultaneous localization and mapping (SLAM) [25] and has aided inertial navigation systems [26] in recent years. Its symmetry ensures that the estimation error satisfies the “log-linear” autonomous differential equation on the Lie algebra of the corresponding Lie group in the system dynamics. Thus, it is possible to design a nonlinear observer or state estimator that exhibits strong convergence within an attraction domain independent of the system’s trajectory. Yu et al. [27] presented a similar approach for wheeled platforms, mainly using speed observations from encoders. We have extended this to legged robot platforms. Unlike wheeled platforms, speed observations from encoders at the foot-end are less reliable than those from wheel encoders. To address this issue, we incorporate the contact point positions of each leg into the filter state variables and consider both foot-end position and velocity observations in the observation equation, making the filter more suitable for legged robots.

The main contributions of this paper are as follows:

1. Model foot-end slippage caused by legged robot motion on non-stationary terrain as a deviation term of body velocity, reducing the drift caused by foot-end slippage through velocity deviation estimation.
2. Develop a real-time RI-EKF state and slip estimator for quadruped robots by fusing foot-end velocity and position observations.
3. Validate the mathematical derivation and the proposed state estimator’s effectiveness through experimental results using the Jueying Mini robot on multiple non-stationary terrains.

## 2. Theoretical Background

We assume a matrix Lie group denoted as G, with its corresponding Lie algebra denoted as g. Let ·^:Rdimg→g represent the isomorphism that maps elements in the tangent space at the identity element to their corresponding matrix representations in G. The exponential mapping of the Lie group is denoted by $\exp:R^{dimg}\to G$ and is represented as exp(ξ)=expm(ξ^), where $\exp_{m}(\cdot )$ is the usual exponential mapping of n×n matrices. A system that evolves over time t on a Lie group can be represented by the dynamics $\frac{d}{dt}X_{t}=f_{u_{t}}\left(X_{t}\right)$, where $X_{t}\in G$ represents the system state and ${\hat{X}}_{t}$ is usually used to denote the estimated state.

**Definition** **1.** (Right-invariant and left-invariant errors). *The right-invariant and left-invariant errors between the two trajectories* $X_{t}$ *and* ${\hat{X}}_{t}$ *are defined as follows:*

(1) $$\eta_{t}^{r}={\hat{X}}_{t}X_{t}^{-1}=\left({\hat{X}}_{t}L\right){\left(X_{t}L\right)}^{-1}\cdot \mathrm{(right-invariant error)}$$

(2) $$\eta_{t}^{l}=X_{t}^{-1}{\hat{X}}_{t}={\left(L{\hat{X}}_{t}\right)}^{-1}\left(LX_{t}\right)\cdot \mathrm{(left-invariant error)}$$

*where* L *is an arbitrary element of the group* G.

**Definition** **2.** (Adjoint map). *Let* G *be a matrix Lie group, with its Lie algebra denoted as* g*. For any* X∈G*, the adjoint map* ${\mathrm{Ad}}_{X}:g\to g$ *is a linear map that satisfies* AdXξ^:Xξ^X−1*. Furthermore, the matrix representation of the adjoint map is denoted by* ${\mathrm{Ad}}_{X}$.

**Theorem** **1.** *A system is group-affine, if for all *t>0*,*$\;X_{1},X_{2}\in G$*, its dynamics *$f_{u_{t}}\left(X_{t}\right)$ *satisfy the following:*

(3) $$f_{u_{t}}\left(X_{1}X_{2}\right)=f_{u_{t}}\left(X_{1}\right)X_{2}+X_{1}f_{u_{t}}\left(X_{2}\right)-X_{1}f_{u_{t}}\left(I_{d}\right)X_{2}$$

*where* $I_{d}\in G$ *denotes the group identity element. If a system is group-affine, then its right-invariant and left-invariant error dynamics are independent of the trajectory and satisfy the following:*

(4) $$\frac{d}{dt}\eta_{t}^{r}=g_{u_{t}}^{r}\left(\eta_{t}^{r}\right),\;\mathrm{where}\;g_{u_{t}}^{r}\left(\eta \right)=f_{u_{t}}\left(\eta \right)-\eta f_{u_{t}}\left(I_{d}\right)$$

(5) $$\frac{d}{dt}\eta_{t}^{l}=g_{u_{t}}^{l}\left(\eta_{t}^{l}\right),\;\mathrm{where}\;g_{u_{t}}^{l}\left(\eta \right)=f_{u_{t}}\left(\eta \right)-f_{u_{t}}\left(I_{d}\right)\eta$$

*We define a matrix* $A_{t}$ *of size* dimg×dimg*, such that for any* t≥0*, the function* gutexp⁡ξ:=Atξ^+Oξ2 *holds. Let* $\xi_{t}$ *be the solution to the following linear differential equation:*

(6) $$\frac{d}{dt}\xi_{t}=A\xi_{t}$$

**Theorem** **2.** *Consider any two trajectories defined by (4) and (5) with a left-invariant and right-invariant error, respectively. For any initial error* $\xi_{0}\in R^{\dim g}$*, if* $\eta_{0}\in exp\left(\xi_{0}\right)$*, then for all* t≥0*, the nonlinear estimation error* $\eta_{t}$ *can be accurately recovered by the linear differential equation (6), where* $\eta_{t}\in exp\left(\xi_{t}\right)$*.*

## 3. System Model

### 3.1. State Equation

Our state estimator can estimate the state variables of the robot’s motion in the world coordinate system $\left\{W\right\}$, which include the robot’s orientation $R_{WB}$, the robot’s position *_W_p_B_*, the robot’s velocity *_W_v_B_*, and the positions of the four feet *_W_p_f_1__*, *_W_p_f_2__*, *_W_p_f_3__*, and *_W_p_f_4__*. To consider the impact of end-effector slippage on state estimation, we use the disturbance observer idea, introducing a bias term $b_{B}^{v}$ modeled as drift velocity and explicitly incorporating it into the state equations. These state variables form a group in ${SE}_{7}\left(3\right)$; hence, we represent the state variables of our InEKF model as follows:

(7) Xt=RWB(t)vBW(t)pBW(t)pf1W(t)pf2W(t)pf3W(t)pf4W(t)bBv(t)07.3I7

Similar to [27], we describe the slippage velocity using an autoregressive model, modeling the slippage velocity as an exponentially decaying variable:

(8) $${\dot{b}}_{B}^{v}(t)=-\alpha b_{B}^{v}(t)+n_{bv}(t),\;n_{bv}(t)∼N\left(0_{3},\Sigma^{b_{B}^{v}}\right)$$

where α>0 denotes the velocity’s decay rate. The IMU provides the following measurements:

(9) $${\tilde{w}}_{t}=w_{t}+n_{t}^{w},\;{\tilde{a}}_{t}=a_{t}+n_{t}^{a}$$

The measurement of the position of the $i_{th}$ foot-end primarily depends on the joint encoder measurements of the $i_{th}$ leg:

(10) $${\tilde{\theta }}_{t}^{f_{i}}=\theta_{t}^{f_{i}}+n_{t}^{f_{i}}$$

Based on the IMU measurement model and the foot-end contact model, the system’s dynamic model is as follows:

(11) $${\dot{R}}_{WB}(t)=R_{WB}(t){\left({\tilde{w}}_{t}-n_{t}^{w}\right)}_{\times }$$

(12) v˙BW(t)=RWB(t)a˜t−nta+g

(13) p˙BW(t)=vBW(t)

(14) p˙fiW(t)=RWB(t)hfi(θ˜tfi)ntfi

where g is the gravity vector, and $h_{f_{i}}\left({\tilde{\theta }}_{t}^{f_{i}}\right)$ is the forward kinematics function which maps the joint position to the relative position from the body coordinate to the $i_{th}$ foot. We can rewrite the above model in matrix form:

(15) ddtXt=RWB(t)[w˜t]×RWB(t)a˜t+gvBW(t)03.103.103.103.1−αbBv(t)07.307.7−Xtnt∧≜futXt−Xtnt∧

where $n_{t}\overset{\Delta }{=}\mathrm{vec}\left(n_{t}^{w},n_{t}^{a},0,h_{f_{1}}\left({\tilde{\theta }}_{t}^{f_{1}}\right)n_{t}^{f_{1}},h_{f_{2}}\left({\tilde{\theta }}_{t}^{f_{2}}\right)n_{t}^{f_{2}},h_{f_{3}}\left({\tilde{\theta }}_{t}^{f_{3}}\right)n_{t}^{f_{3}},h_{f_{4}}\left({\tilde{\theta }}_{t}^{f_{4}}\right)n_{t}^{f_{4}}\right)$. It can be proven that the deterministic system dynamics $f_{u_{t}}\left(\cdot \right)$ satisfy the group affine property (3). According to Theorem 1, the left-invariant and right-invariant error dynamics are autonomous and evolve independently of the system’s state. The system’s right-invariant error is as follows:

(16) $$\begin{array}{c} \frac{d}{dt}\eta_{t}^{r}=f_{u_{t}}\left(\eta_{t}^{r}\right)-\eta_{t}^{r}f_{u_{t}}\left(I_{d}\right)+\left({\hat{X}}_{t}n_{t}^{\wedge }{\hat{X}}_{t}^{-1}\right)\eta_{t}^{r}\\ :=g_{u_{t}}^{r}\left(\eta_{t}^{r}\right)+{\left({\mathrm{Ad}}_{\hat{X}}n_{t}\right)}^{\wedge }\eta_{t}^{r} \end{array}$$

Theorem 2 further explains the logarithmic linear property of invariant errors. If $A_{t}$ is defined as

(17) $$g_{u_{t}}^{r}\left(\exp(\xi )\right)≜{\left(A_{t}\xi \right)}^{\wedge }+O\left({\left\|\xi \right\|}^{2}\right)$$

then the invariant error ξ satisfies the following linear system:

(18) $$\frac{d}{dt}\xi_{t}=A_{t}\xi_{t}+{\mathrm{Ad}}_{\hat{X}}n_{t}$$

Using first-order approximation to linearize $g_{u_{t}}^{r}\left(\eta_{t}^{r}\right)$ yields the following:


(19) ηtr=exp(ξt)≈Id+ξt^

Substituting this into the expression for $g_{u_{t}}^{r}\left(\eta_{t}^{r}\right)$, the following equation is obtained:

(20) $$g_{u_{t}}^{r}\left(\eta_{t}^{r}\right)={\left(\left[\begin{array}{cccc} 0_{3.3} & 0_{3.3} & 0_{3.15} & 0_{3.3}\\ {\left[g\right]}_{\times } & 0_{3.3} & 0_{3.15} & 0_{3.3}\\ 0_{3.3} & I_{3} & 0_{3.15} & 0_{3.3}\\ 0_{12.3} & 0_{12.3} & 0_{12.15} & 0_{12.3}\\ 0_{3.3} & 0_{3.3} & 0_{3.15} & -\alpha I_{3} \end{array}\right]\xi_{t}\right)}^{\wedge }$$

Based on these computations, the prediction step of the right-invariant extended Kalman filter (EKF) can be derived. The state estimate is denoted as ${\hat{X}}_{t}$, and the covariance matrix $P_{t}$ is computed using the Riccati equation:

(21) $$\frac{d}{dt}{\hat{X}}_{t}=f_{u_{t}}\left({\hat{X}}_{t}\right)$$

(22) $$\frac{d}{dt}P_{t}=A_{t}P_{t}+P_{t}A_{t}^{Τ}+{\bar{Q}}_{t}$$

where ${\bar{Q}}_{t}={\mathrm{Ad}}_{\hat{X}}\mathrm{Cov}(n_{t}){\mathrm{Ad}}_{\hat{X}}$.

### 3.2. Measurement Model

According to the forward kinematics of the quadruped robot, the position of the foot relative to the body coordinate system {B} is given by the following:

(23) pfiB(t)=hfiθtfi=RWBT(t)pfiW(t)−pBW(t)

The velocity of the foot is composed of the velocity generated by the joint rotation vbjfiB and the velocity of the body motion vbefiB

(24) vbjfiB(t)=Jiθ˙tfi

(25) vbefiB(t)=vBB(t)+wt×pfiB(t)

where $J_{i}$ is the Jacobian matrix of the $i_{th}$ leg. By transforming vbjfiB and vbefiB into the world coordinate system {W}, we can obtain the foot velocity in the world coordinate system:

(26) vfiW(t)=RWB(t)vbjfiB(t)+vbefiB(t)=vBW(t)+RWB(t)Jiθ˙tfi+wt×pfiB(t)

When the foot is in stable contact with the ground without slipping, vfiW(t)=0, that is

(27) vBW(t)=−RWB(t)Jiθ˙tfi+wt×pfiB(t).

Assuming that the joint encoder is affected by Gaussian white noise with a covariance matrix of $R_{\theta }$, i.e., ${\tilde{\theta }}_{t}=\theta_{t}+n_{t}^{\theta }$, we can derive the following observation model:

(28) hfi(θ˜tfi)=RWBΤ(t)pfiW(t)−pBW(t)+Jiθ˜tfintθ

(29) vBW(t)=−RWB(t)Jiθ˜˙tfi+wt×hfi(θ˜tfi)+R˙WB(t)Jiθ˜tfintθ

Therefore, when in stable contact, the following two observations exist:

(30) $$\left[\begin{array}{c} h_{f_{i}}({\tilde{\theta }}_{t}^{f_{i}})\\ 0\\ 1\\ \begin{array}{l} 0_{i-1.1}\\ -1\\ 0_{5-i,1} \end{array} \end{array}\right]=X_{t}^{-1}\left[\begin{array}{l} 0_{3.1}\\ 0\\ 1\\ 0_{i-1.1}\\ -1\\ 0_{5-i,1} \end{array}\right]+\left[\begin{array}{c} J_{i}{\tilde{\theta }}_{t}^{f_{i}}n_{t}^{\theta }\\ 0_{6.1} \end{array}\right],\;\left[\begin{array}{c} -\left(J_{i}{\dot{\tilde{\theta }}}_{t}^{f_{i}}+w\times h_{f_{i}}({\tilde{\theta }}_{t}^{f_{i}})\right)\\ -1\\ 0_{5.1} \end{array}\right]=X_{t}^{-1}\left[\begin{array}{c} 0_{3.1}\\ -1\\ 0_{5.1} \end{array}\right]+\left[\begin{array}{c} {\dot{R}}_{WB}(t)J_{i}{\tilde{\theta }}_{t}^{f_{i}}n_{t}^{\theta }\\ 0_{6.1} \end{array}\right],\;i=1,2,\ldots ,4$$

The first equation represents the observation of the foot position of the quadruped robot, denoted as $Y_{t}^{p_{f_{i}}}=X_{t}^{-1}c_{t}^{p_{f_{i}}}+N_{t}^{p_{f_{i}}}$ where, $Y_{t}^{p_{f_{i}}}={\left[\begin{array}{cccccc} h_{f_{i}}^{Τ}({\tilde{\theta }}_{t}^{f_{i}}) & 0 & 1 & 0_{1,i-1} & -1 & 0_{1.5-i} \end{array}\right]}^{Τ}$,$c^{p_{f_{i}}}={\left[\begin{array}{cccccc} 0_{1.3} & 0 & 1 & 0_{1,i-1} & -1 & 0_{1.5-i} \end{array}\right]}^{Τ}$, $N_{t}^{p_{f_{i}}}={\left[\begin{array}{cc} {\left(J_{i}{\tilde{\theta }}_{t}^{f_{i}}n_{t}^{\theta }\right)}^{Τ} & 0_{1.6} \end{array}\right]}^{Τ}$, and i=1,2,…,4. The second equation represents the observation of the body velocity of the quadruped robot, denoted as $Y_{t}^{v_{i}}=X_{t}^{-1}c^{v_{i}}+N_{t}^{v_{f_{i}}}$, where $Y_{t}^{v_{i}}={\left[\begin{array}{ccc} -\left(J_{i}{\dot{\tilde{\theta }}}_{t}^{f_{i}}+w_{t}\times h_{f_{i}}({\tilde{\theta }}_{t}^{f_{i}})\right) & -1 & 0_{1.5} \end{array}\right]}^{Τ}$, $c^{v}={\left[\begin{array}{ccc} 0_{1.3} & -1 & 0_{1.5} \end{array}\right]}^{Τ}$, $N_{t}^{v_{f_{i}}}={\left[\begin{array}{cc} {\left({\dot{R}}_{WB}(t)J_{i}{\tilde{\theta }}_{t}^{f_{i}}n_{t}^{\theta }\right)}^{Τ} & 0_{1.6} \end{array}\right]}^{Τ}$, and i=1,2,…,4. Hartley [10] used position as the observation in bipedal robots, while Teng [16] used velocity as the observation. In practice, these two observations can be considered together. Using the moving pose as an example, the observation error is defined as follows:

(31) $$Z_{t}^{p_{f_{i}}}={\hat{X}}_{t}Y_{t}^{p_{f_{i}}}-c_{t}^{p_{f_{i}}}$$

Substituting $Y_{t}^{p_{f_{i}}}=X_{t}^{-1}c_{t}^{p_{f_{i}}}+N_{t}^{p_{f_{i}}}$ into (31), it can be noted that

(32) $$\begin{array}{c} Z_{t}^{p_{f_{i}}}={\hat{X}}_{t}\left(X_{t}^{-1}c_{t}^{p_{f_{i}}}+N_{t}^{p_{f_{i}}}\right)-c_{t}^{p_{f_{i}}}\\ =\eta_{t}c_{t}^{p_{f_{i}}}-c_{t}^{p_{f_{i}}}+{\hat{X}}_{t}N_{t}^{p_{f_{i}}}\\ =\left[\begin{array}{c} \xi_{t}^{p}-\xi_{t}^{p^{f_{i}}}\\ 0_{7.1} \end{array}\right]+\left[\begin{array}{c} {\hat{R}}_{t}J_{i}{\tilde{\theta }}_{t}^{f_{i}}n_{t}^{\theta }\\ 0_{7.1} \end{array}\right] \end{array}.$$

Note that for each foot’s data observation, except for the first row, all other elements are zero. Let $z_{t}^{p_{f_{i}}}$ be the term $Z_{t}^{p_{f_{i}}}$ where all zero elements are removed:

(33) $$z_{t}^{p_{f_{i}}}=\left[\begin{array}{cccccc} 0_{3.3} & 0_{3.3} & I_{3} & 0_{3.3*(i-1)} & -I_{3} & 0_{3.3*(5-i)} \end{array}\right]\xi_{t}-{\hat{R}}_{WB}(t)J_{i}{\tilde{\theta }}_{t}^{f_{i}}n_{t}^{\theta }$$

Combining all foot position observations, we obtain the following:

(34) $$\left[\begin{array}{c} z_{t}^{p_{f_{1}}}\\ z_{t}^{p_{f_{2}}}\\ z_{t}^{p_{f_{3}}}\\ z_{t}^{p_{f_{4}}} \end{array}\right]=\left[\begin{array}{cccccccc} 0_{3.3} & 0_{3.3} & I_{3} & -I_{3} & 0_{3.3} & 0_{3.3} & 0_{3.3} & 0_{3.3}\\ 0_{3.3} & 0_{3.3} & I_{3} & 0_{3.3} & -I_{3} & 0_{3.3} & 0_{3.3} & 0_{3.3}\\ 0_{3.3} & 0_{3.3} & I_{3} & 0_{3.3} & 0_{3.3} & -I_{3} & 0_{3.3} & 0_{3.3}\\ 0_{3.3} & 0_{3.3} & I_{3} & 0_{3.3} & 0_{3.3} & 0_{3.3} & -I_{3} & 0_{3.3} \end{array}\right]\xi_{t}+\left[\begin{array}{c} {\hat{R}}_{t}J_{1}{\tilde{\theta }}_{t}^{f_{1}}n_{t}^{\theta }\\ {\hat{R}}_{t}J_{2}{\tilde{\theta }}_{t}^{f_{2}}n_{t}^{\theta }\\ {\hat{R}}_{t}J_{3}{\tilde{\theta }}_{t}^{f_{3}}n_{t}^{\theta }\\ {\hat{R}}_{t}J_{4}{\tilde{\theta }}_{t}^{f_{4}}n_{t}^{\theta } \end{array}\right]$$

Similarly, for velocity observations, we obtain the following:

(35) $$\left[\begin{array}{c} z_{t}^{v_{1}}\\ z_{t}^{v_{2}}\\ z_{t}^{v_{3}}\\ z_{t}^{v_{4}} \end{array}\right]=\left[\begin{array}{ccc} 0_{3.3} & I_{3} & 0_{3.18}\\ 0_{3.3} & I_{3} & 0_{3.18}\\ 0_{3.3} & I_{3} & 0_{3.18}\\ 0_{3.3} & I_{3} & 0_{3.18} \end{array}\right]\xi_{t}+\left[\begin{array}{c} {\left[{\tilde{w}}_{t}\right]}_{\times }J_{1}{\tilde{\theta }}_{t}^{f_{1}}n_{t}^{\theta }\\ {\left[{\tilde{w}}_{t}\right]}_{\times }J_{2}{\tilde{\theta }}_{t}^{f_{2}}n_{t}^{\theta }\\ {\left[{\tilde{w}}_{t}\right]}_{\times }J_{3}{\tilde{\theta }}_{t}^{f_{3}}n_{t}^{\theta }\\ {\left[{\tilde{w}}_{t}\right]}_{\times }J_{4}{\tilde{\theta }}_{t}^{f_{4}}n_{t}^{\theta } \end{array}\right]$$

By merging velocity observations with position observations, this leads to the following:

(36) $$\left[\begin{array}{c} z_{t}^{p_{f_{1}}}\\ z_{t}^{p_{f_{2}}}\\ z_{t}^{p_{f_{3}}}\\ z_{t}^{p_{f_{4}}}\\ z_{t}^{v_{1}}\\ z_{t}^{v_{2}}\\ z_{t}^{v_{3}}\\ z_{t}^{v_{4}} \end{array}\right]=\left[\begin{array}{cccccccc} 0_{3.3} & 0_{3.3} & I_{3} & -I_{3} & 0_{3.3} & 0_{3.3} & 0_{3.3} & 0_{3.3}\\ 0_{3.3} & 0_{3.3} & I_{3} & 0_{3.3} & -I_{3} & 0_{3.3} & 0_{3.3} & 0_{3.3}\\ 0_{3.3} & 0_{3.3} & I_{3} & 0_{3.3} & 0_{3.3} & -I_{3} & 0_{3.3} & 0_{3.3}\\ 0_{3.3} & 0_{3.3} & I_{3} & 0_{3.3} & 0_{3.3} & 0_{3.3} & -I_{3} & 0_{3.3}\\ 0_{3.3} & I_{3} & 0_{3.3} & 0_{3.3} & 0_{3.3} & 0_{3.3} & 0_{3.3} & 0_{3.3}\\ 0_{3.3} & I_{3} & 0_{3.3} & 0_{3.3} & 0_{3.3} & 0_{3.3} & 0_{3.3} & 0_{3.3}\\ 0_{3.3} & I_{3} & 0_{3.3} & 0_{3.3} & 0_{3.3} & 0_{3.3} & 0_{3.3} & 0_{3.3}\\ 0_{3.3} & I_{3} & 0_{3.3} & 0_{3.3} & 0_{3.3} & 0_{3.3} & 0_{3.3} & 0_{3.3} \end{array}\right]\xi_{t}+\left[\begin{array}{c} {\hat{R}}_{t}J_{1}{\tilde{\theta }}_{t}^{f_{1}}n_{t}^{\theta }\\ {\hat{R}}_{t}J_{2}{\tilde{\theta }}_{t}^{f_{2}}n_{t}^{\theta }\\ {\hat{R}}_{t}J_{3}{\tilde{\theta }}_{t}^{f_{3}}n_{t}^{\theta }\\ {\hat{R}}_{t}J_{4}{\tilde{\theta }}_{t}^{f_{4}}n_{t}^{\theta }\\ {\left[{\tilde{w}}_{t}\right]}_{\times }J_{1}{\tilde{\theta }}_{t}^{f_{1}}n_{t}^{\theta }\\ {\left[{\tilde{w}}_{t}\right]}_{\times }J_{2}{\tilde{\theta }}_{t}^{f_{2}}n_{t}^{\theta }\\ {\left[{\tilde{w}}_{t}\right]}_{\times }J_{3}{\tilde{\theta }}_{t}^{f_{3}}n_{t}^{\theta }\\ {\left[{\tilde{w}}_{t}\right]}_{\times }J_{4}{\tilde{\theta }}_{t}^{f_{4}}n_{t}^{\theta } \end{array}\right]$$

### 3.3. Considering Unstable Contact and Slipping

The above model assumes that all four feet are in stable contact with the ground. In practice, however, the quadruped robot’s feet may be airborne during walking, and on many uneven or slippery terrains, the feet may make unstable contact. When the feet are airborne or in unstable contact, the foot positions calculated by the state space model do not conform to this assumption. To address this issue, we use the foot contact probability model proposed in [9] to determine the probability of stable contact between the feet and the ground:

(37) $$P_{k}\left(S_{i}=1|f_{k}^{i}\right)=\frac{1}{1+\exp\left(-\beta_{1}f_{z,i}^{k}-\beta_{2}\right)}$$

where $\beta_{1}$ and $\beta_{2}$ are constants. $f_{k}^{i}$ represents the reaction force exerted on the foot-end of the $i_{th}$ leg by the ground at time k and $f_{z,i}^{k}$ represents the z-axis component of the this reaction force. $S_{i}=1$ indicates that the foot-end of the $i_{th}$ leg is in stable contact with the ground. Using this contact probability model, we can combine the basic velocities generated by each leg to obtain the body’s velocity and use it as an observation of the body’s velocity:

(38) vBB=∑i∈CPkSi=1|fkip˙fiB∑i∈CPkSi=1|fki

where C is the set of feet that exceed the 0.5 threshold of the logistic regression. Considering the velocity bias, the velocity expression of the quadruped robot in the world coordinate system at time t is as follows:

(39) vBW(t)=RWB(t)vBB(t)+bBv(t)+ntv

where $n_{t}^{v}$ represents the error in the body’s velocity, which includes the error generated by each foot’ velocity observation. To correctly compute the covariance matrix $\sum_{t}^{v}$ of $n_{t}^{v}$, we consider the consistency between each foot’s velocity p˙fiB and the ground’s impact. For each axis $r\in \left\{x,y,z\right\}$, the variance at a given moment can be computed as follows:

(40) σr2(k)=σ02+γ1stdp˙fi∈CB(k)C+(1−γ1)γ2Δf¯zk2

where $\left|∆{\bar{f}}_{z}\right|=\frac{1}{dim(C)}\sum_{i\in C}\left|f_{z,i}^{k}-f_{z,i}^{k-1}\right|$ is the average absolute difference in the ground reaction force in the *z* axis between the current and previous contact times. $\sigma_{0}^{2}$ is the baseline standard deviation of velocity, while stdp˙fi∈CB is the standard deviation of the stance-phase foot velocity contribution for the $i_{th}$ elements. $\gamma_{1}$ is a factor that balances consistency and the impact of collision forces (we use 0.5), while $\gamma_{2}$ is a normalization factor to normalize the typical velocity error difference $\left|∆{\bar{f}}_{z}\right|$ at the same moment.

For the observation of foot position, since the foot may be in a hovering state or in unstable contact, the estimated foot position obtained through the state space model might be unreliable. By dynamically adjusting the noise covariance of the foot contact using the formula below, the impact of unstable or non-contact states on state estimation can be mitigated:

(41) $$\Sigma_{t}^{p_{f_{i}}}\left(k\right)=\left(1+L\left(1-P_{k}\left(S_{i}=1|f_{k}^{i}\right)\right)\right)\Sigma_{t}^{p_{f_{i}}}\left(k\right)$$

where *L* is a sufficiently large scalar. The final observation model accounting for unstable contact and sliding is as follows:

(42) $$\left[\begin{array}{c} z_{t}^{p_{f_{1}}}\\ z_{t}^{p_{f_{2}}}\\ z_{t}^{p_{f_{3}}}\\ z_{t}^{p_{f_{4}}}\\ z_{t}^{v} \end{array}\right]=\left[\begin{array}{cccccccc} 0_{3.3} & 0_{3.3} & I_{3.3} & -I_{3.3} & 0_{3.3} & 0_{3.3} & 0_{3.3} & 0_{3.3}\\ 0_{3.3} & 0_{3.3} & I_{3.3} & 0_{3.3} & -I_{3.3} & 0_{3.3} & 0_{3.3} & 0_{3.3}\\ 0_{3.3} & 0_{3.3} & I_{3.3} & 0_{3.3} & 0_{3.3} & -I_{3.3} & 0_{3.3} & 0_{3.3}\\ 0_{3.3} & 0_{3.3} & I_{3.3} & 0_{3.3} & 0_{3.3} & 0_{3.3} & -I_{3.3} & 0_{3.3}\\ 0_{3.3} & I_{3.3} & 0_{3.3} & 0_{3.3} & 0_{3.3} & 0_{3.3} & 0_{3.3} & 0_{3.3} \end{array}\right]\xi_{t}+\left[\begin{array}{c} n_{t}^{p_{f_{1}}}\\ n_{t}^{p_{f_{2}}}\\ n_{t}^{p_{f_{3}}}\\ n_{t}^{p_{f_{4}}}\\ n_{t}^{v} \end{array}\right]$$

An observation equation of the form $z_{t}=C\xi_{t}+V_{t}$ is obtained. Based on the definition of a right-invariant error, the update steps for the InEKF are as follows:

(43) $$K_{t}=P_{t}C_{t}^{T}\left(C_{t}P_{t}C_{t}^{T}+N_{t}\right)$$

(44) $$X_{t}^{+}=\mathrm{Exp}\left(K_{t}z_{t}\right){\hat{X}}_{t}$$

(45) $$P_{t}^{+}=\left(I-K_{t}C_{t}\right)P_{t}$$

## 4. Experimental Results and Analysis

**Figure 1:**
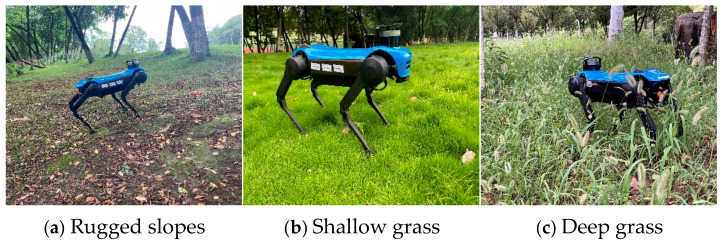
Test environments.

To validate the effectiveness of the proposed algorithm, experiments were conducted on three different terrains using the Jueying Mini quadruped robot. The Jueying Mini quadruped robot is equipped with an IMU and 12 joint encoders, all with a measurement frequency of 166 Hz. The IMU provides measurements of angular velocity and linear acceleration, while the joint encoders provide measurements of joint angles, joint torques, and joint angular velocities. The initial values and parameters of the filter are as follows: $R_{WB}(0)=I_{3}$, *_W_v_B_*(0) = [0 0 0]*^T^*, *_W_p_B_*(0) = [0 0 0.2]*^T^*, *_W_p_f_i__*(0) = *_W_p_B_*(0) + *h_f_i__*($\theta_{0}^{f_{i}}$), *α* = 20, *P* = *I*_24_ and *L* = 10^4^. Additionally, Table 1 presents the corresponding noise parameters. The experimental terrains include typical outdoor rugged slopes, shallow grass, and deep grass, as shown in Figure 1.

### 4.1. Contact Probability Calculation

We use the phase information of the foot-end as the true value of the contact state between the foot-end and the ground to calculate the parameters $\beta_{1}$ and $\beta_{2}$ in Equation (37). The ground reaction force on the foot-end is solved by the joint torque:

(46) fki=−JiTθtfiτi−hi(θtfi,θ˙tfi,gB)

**Figure 2:**
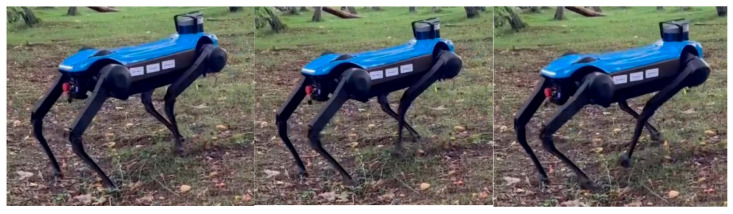
Foot slipping scenarios of a quadruped robot during ground contact.

**Figure 3:**
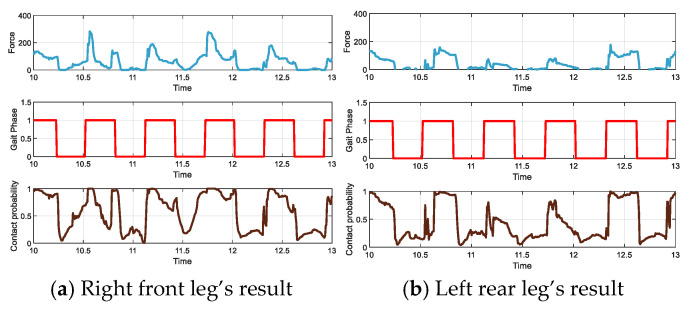
Estimation of foot contact probability during unstable contact events, with (**a**) representing right front leg and (**b**) left rear leg.

Considering that the foot-end starts to touch the ground and prepares to leave the ground, the position of the foot-end always undergoes some displacement. An appropriate adjustment of the parameter β reduces the sensitivity of the model to input variation, thereby making the fluctuation in the foot-end contact probability smoother. In our experiment, ***β*_1_ = 1.3** and ***β*_2_ = 0.037**. Figure 2 illustrates the scenario where the quadruped robot’s foot slips during contact with the ground, and Figure 3 presents the final results, showing the probability model for right-front-foot slipping.

### 4.2. Algorithm Evaluation

**Figure 4:**
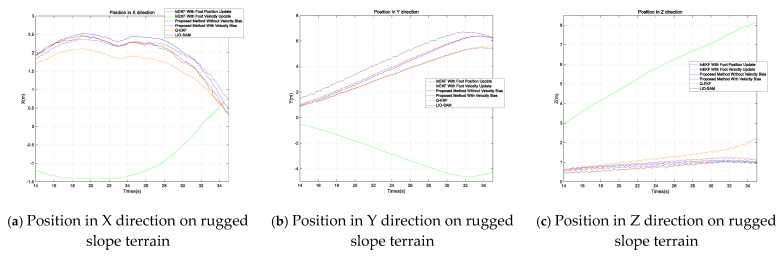

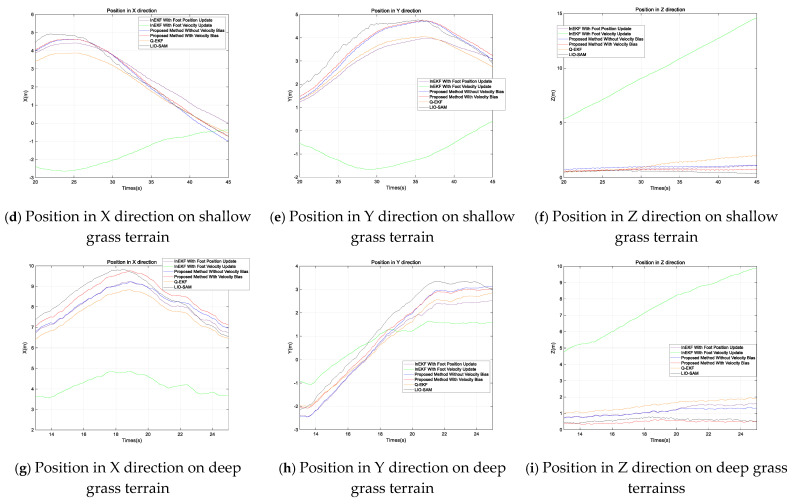
The position estimates of the quadruped robot in the X, Y, and Z directions on different terrains, with (**a**–**c**) depicting the position estimate for rugged slope terrain, (**d**–**f**) for shallow grass terrain, and (**g**–**i**) for deep grass terrain.

**Figure 5:**
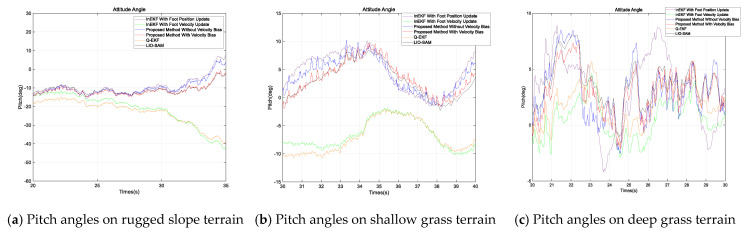

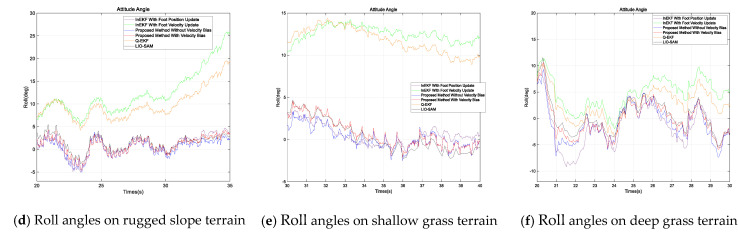
Pitch and roll angle estimation of the quadruped robot on different terrains, with (**a**,**d**) depicting the estimate for rugged slope terrain, (**b**,**e**) for shallow grass terrain, and (**c**,**f**) for deep grass terrain.

Due to the absence of a motion capture system in outdoor areas, we used the LiDAR SLAM method, which performs relatively well in outdoor environments, with LIO-SAM’s computed pose information serving as the ground truth. The experimental results are shown in the figures. Figure 4 presents the position estimate results of various methods for the quadruped robot across three different terrains, with the position information in each case being provided for the X, Y, and Z directions. Figure 5 presents the attitude angle estimates; as the yaw is unobservable [10], only the pitch and roll angles are presented. We compared a total of five methods: the traditional quaternion-based Kalman filter method (Q-EKF) [8], the InEKF with only velocity updates [16], the InEKF with only position updates, and the proposed method considering and not considering velocity bias. For the methods [10,16], when the foot is in the contact phase, it is determined to be in stable contact with the ground, while the remaining methods employ the proposed contact probability judgment and covariance adjustment strategy. The results from the figure show that the proposed method is closer to the true trajectory.

In Figure 4, it is evident that the InEKF with only velocity updates exhibits significant deviations in position estimation. One possible reason is that the measurement of velocity itself has a relatively large deviation. After multiple filtrations, the accumulated error progressively increases and eventually diverges. In the method presented in reference [16], the author achieved relatively accurate results through fusion with camera observations. However, in the method of this paper, only proprioceptive sensors are discussed. When reproducing the method of [16], camera observations are not incorporated. Consequently, a large deviation occurs, which also indicates that the deviation in foot-end velocity measurement is indeed real.

As depicted in Figure 5, it is apparent that the attitude angles estimated by the two methods, the Q-EKF and the InEKF with only velocity updates, deviate significantly from the true values. The inaccuracy of the InEKF with only velocity updates has been previously addressed, potentially stemming from the deviation in velocity measurement. In contrast, the other methods are relatively close to the actual values, indicating that the method based on the InEKF is better than the Q-EKF in the estimation of attitude angles.

The trajectories of different methods under various terrains were evaluated using the EVO tool [28], with the results shown in Table 2. The evaluation metrics include the Absolute Trajectory Error (ATE) and the Relative Pose Error (RPE), where ATE and RPE measure position error (in meters) and rotation error (in radians), respectively.

Table 2 shows that the proposed method (PM with Vel Bias) outperforms other methods in ATE and RPE across all terrains, especially on rugged slopes and shallow grass, where its position error (ATE) and attitude error (RPE) are the lowest, at 0.4572 m and 0.0345 radians (for rugged slope position error) and 0.3431 m and 0.0367 radians (for shallow grass position error), respectively. This indicates that this method offers higher accuracy and robustness in scenarios with velocity bias compensation. In contrast, InEKF with Vel update shows larger errors in most terrains, particularly in deep grass, where the ATE reaches 3.3818 m and the RPE is 1.3146 radians, demonstrating its sensitivity to errors in complex terrains. However, the method shows good stability and accuracy in RPE rotational error performance across various terrains, particularly on shallow grass and rugged slopes. These results indicate that velocity observation plays a crucial role in maintaining low rotational errors. Combining velocity observations with other methods (such as position observation) holds promise for achieving more robust attitude estimation. PM without Vel Bias also shows relatively stable performance in shallow grass and deep grass, especially in position error, suggesting that the strategy of removing velocity bias can be beneficial for trajectory estimation accuracy under certain terrain conditions. The QEKF method’s performance ranks between the other methods but has the lowest rotation error (RPE) on shallow grass, at 0.0367 radians, indicating its advantage in handling certain types of attitude changes. Overall, the experimental results show that the proposed method provides the best performance in most cases.

## 5. Conclusions

To address the state estimation drift issue caused by unstable foot contact in non-stationary terrains for quadruped robots, we propose an improved invariant extended Kalman filter (InEKF) method. This method models foot-end sliding as a bias term in body velocity and integrates both foot-end velocity and position observations into the observation equation. By using foot-end contact probability assessment and adaptive covariance adjustment strategies, it effectively improves the state estimation accuracy of quadruped robots in complex outdoor environments. The experimental results show that compared to several existing methods (such as Q-EKF and InEKF with a single observation update), our method exhibits a lower Root Mean Square Error (RMSE) across multiple non-stationary terrains and demonstrates significant advantages in position and attitude estimation. This method offers a new approach to the state estimation problem of quadruped robots on non-stationary terrains and suggests further optimization of the filtering algorithm for application in more complex environments in the future.

Future research directions could focus on several aspects: firstly, investigating the potential of fusion with external perception sensors, such as vision cameras, lidars, etc., to enhance the robot’s positioning and mapping capabilities; secondly, exploring the adaptation of this filtering algorithm to handle more extreme and unpredictable terrains, including those with rapidly changing surface properties or dynamic obstacles; thirdly, studying the possibility of optimizing the computational efficiency of the algorithm to enable real-time state estimation with lower latency, especially in scenarios where the quadruped robot needs to make quick decisions based on its current state.

## Figures and Tables

**Table 1:** Noise parameters and initial covariances.

Measurement Type	Noise Std. Dev	State Variable	Initial Covariance
Gyroscope	0.1 rad/s	Robot Orientation	0.03 rad
Accelerometer	0.1 m/s^2^	Robot Velocity	0.01 m/s
Foot Encoder Pos	0.01 m	Robot Position	0.01 m
Foot Encoder Vel	0.1 m/s	Robot Slip Velocity	0.01 m/s
Disturbance Process	5 m/s		

**Table 2:** RMSE evaluation of different state estimation methods for quadruped robots on various terrains.

Terrain	Method	ATE RMSE		RPE RMSE	
		Position (m)	Rotation (rad)	Position (m)	Rotation (rad)
Rugged Slopes	QEKF	1.3459	1.0541	0.0654	0.0385
	InEKF with Vel update	1.5898	2.8388	0.0743	0.0395
	InEKF with Pos update	0.4704	0.2185	0.0633	0.0358
	PM without Vel Bias	0.4666	0.1628	0.0601	0.0358
	PM with Vel Bias	**0.4572**	**0.1626**	**0.0601**	**0.0345**
Shallow Grass	QEKF	0.7893	0.4661	0.0697	**0.0367**
	InEKF with Vel update	2.3747	3.0528	0.0665	0.0368
	InEKF with Pos update	0.6525	0.3039	0.0626	0.0655
	PM without Vel Bias	0.3501	**0.1238**	0.0393	0.0630
	PM with Vel Bias	**0.3431**	0.1261	**0.0391**	0.0641
Deep Grass	QEKF	0.5993	0.2867	0.0628	0.0558
	InEKF with Vel update	3.3818	1.3146	0.0797	0.0452
	InEKF with Pos update	0.7552	0.3526.	0.0653	0.0522
	PM without Vel Bias	0.6920	0.3228	0.0646	0.0506
	PM with Vel Bias	**0.5289**	**0.2035**	**0.0601**	**0.0498**

## Data Availability

The data presented in this study are available on request from the corresponding author.
